# STK11 mutation impacts CD1E expression to regulate the differentiation of macrophages in lung adenocarcinoma

**DOI:** 10.1002/iid3.958

**Published:** 2023-07-27

**Authors:** Qingfeng Zhang, Juan Feng, Kui Liu, Xiaoyan Yang, Yun Huang, Bo Tang

**Affiliations:** ^1^ Department of Cardio‐Thoracic Surgery Zigong Fourth People's Hospital Zigong China; ^2^ Department of Operating Room Zigong Fourth People's Hospital Zigong China

**Keywords:** CD1E, lung adenocarcinoma, macrophages, proliferation, STK11 mutation

## Abstract

**Background:**

The deficiency of serine/threonine protein kinase 11 (STK11), one of the most common tumor suppressor genes in non‐small‐cell lung cancer, is a crucial player in tumor immune microenvironment regulation. This study attempted to unveil how mutated STK11 impact the differentiation of macrophages in lung adenocarcinoma (LUAD).

**Methods:**

STK11 and CD1E expression levels in different cell models were assessed by quantitative reverse transcription polymerase chain reaction. Western blot was utilized to detect the protein expression levels of STK11, CD1E, apoptosis markers, and AMPK signaling pathway markers after transfection treatment. Cell viability and macrophage differentiation were detected by CCK‐8 and flow cytometry. Immunohistochemistry and immunofluorescence were employed to detect the expression of related genes and macrophage markers, respectively.

**Results:**

This study found that STK11 mutations promoted the proliferation of LUAD cells and inhibited the differentiation of M1 macrophages, apoptosis, and the AMPK signaling pathway. Mutated STK11 led to CD1E downregulation, which curbed the differentiation of M1 macrophages and hence promoted LUAD progression. It was further validated by the in vivo experimental results that STK11 mutation significantly decreased the immune infiltration of M1 macrophages and promoted LUAD progression.

**Conclusion:**

It was revealed that STK11 mutation affected CD1E expression to regulate macrophage differentiation in LUAD and then promote tumor progression. In this way, CD1E could be a potential biological target for the therapeutic interventions of STK11‐mutant LUAD patients. These findings also threw new light on a new therapeutic strategy for STK11‐mutant tumor patients that assisted the macrophage polarization pathway.

## Graphical abstract

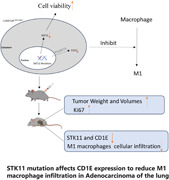

## 1 INTRODUCTION

Lung adenocarcinoma (LUAD) is one of the biggest killers of cancer‐related deaths.^1^ Genetic testing technique advances made in the past decade have enabled the identification of many oncogenes in lung cancer, accompanying diagnostic and therapeutic leaps for advanced lung cancer. Mutations of Kirsten rat sarcoma viral oncogene (KRAS) (32%) and epidermal growth factor receptor (EGFR) are two major risk factors for LUAD.^2^ Compared with conventional chemotherapy or radiotherapy, molecularly targeted therapy yields better results for patients with LUAD. EGFR‐mutated LUAD patients benefit from EGFR tyrosine kinase inhibitors,^3^, ^4^, ^5^ and KRAS patients benefit from allele‐specific KRASG12C inhibitors.^6^, ^7^, ^8^, ^9^ Though considerable survival benefits have been realized by a number of target inhibitors on many lung cancer patients, it remains inaccessible to some patients. Immunotherapy somehow filled the void by providing cancer patients with a novel treatment choice.^10^ By aiding the immune system, immunotherapy enhances its ability to react to tumor antigens and hence facilitates the control and destruction of tumor cells.^11^ The result of immunotherapy varies since tumor immune surveillance and response also vary with the tumor microenvironment (TME) of different cellar components caused by heterogeneity. Therefore, an in‐depth analysis of the association between the pathological mechanism of LUAD and immune cell infiltration in the TME is crucial. This study attempted to provide such an analysis to develop new strategies for LUAD treatment.

The tumor immune microenvironment (TIME) is characterized largely by interactions that occur between different immune cell populations within a tumor. New insights provided by recent studies throw light on the complex mutual relationship between tumor and host immune cells, which are the main factors determining the prognosis of patients, including natural killer (NK) cells, macrophages, lymphocytes, neutrophils, dendritic cells, and myeloid‐derived suppressor cells.^12^ Among them, macrophages infiltrating into TIME or tumor‐associated macrophages (TAM)^13^ that inhibit immune responses and promote tumor growth, are considered to be a tumor‐promotion factor.^13^, ^14^ Macrophages are innate immune cells with two functional phenotypes. Activated lipopolysaccharide‐induced M1 macrophages can generate high expressions of pro‐inflammatory cytokines. Interleukin‐4‐induced M2 macrophages facilitate the expression of various anti‐inflammatory cytokines. In recent years, how TAM is involved in tumor progression via various mechanisms has been widely investigated. For instance, in LUAD, the knockdown of NOD2 induces a tumor‐promoting macrophage phenotype that promotes malignant progression.^12^ Jang et al.^15^ also reported that vildagliptin, a CD26/DPP4 inhibitor, inhibits lung cancer by regulating the cell viability of NK via macrophage. M1 macrophage‐derived exosomes lead to reduced LUAD activity and increased apoptosis via the miR‐181a‐5p/ETS1/STK16 axis.^16^ Relevant clinical studies have confirmed that M1 macrophages are involved in immune surveillance. For instance, non‐small‐cell lung cancer (NSCLC) patients with better survival rates are found to have increased M1 macrophages infiltration in tumor islets and low M2 macrophages infiltration in tumors.^17^ However, M2 macrophages in TIME are found strongly related to the poor prognosis of a variety of cancer patients. In NSCLC, M2 macrophages infiltration into tumor islets contributes to poor prognosis.^18^ Therefore, investigating how TAM impacts tumor development could pave the way for emerging TAM‐targeting therapy.

In LUAD, the serine/threonine protein kinase 11 gene (STK11) is the third most frequently mutated gene that has been identified in 33% of primary LUAD.^19^ STK11 is a tumor suppressor gene that involves in cellular energy regulation, proliferation, apoptosis, and other biological functions.^20^ The role STK11 played in tumors has received much attention. Studies have reported that STK11 mutations bear some relation with poor prognosis in patients with cervical cancer, epithelial ovarian cancer, lung cancer, and so forth.^20^, ^21^ Skoulidis et al.^22^ demonstrated that STK11 mutations are involved in tumor immunotherapy resistance and immune cell infiltration. Besides, according to the results of KRAS‐mutant LUAD development in mice in previous studies, STK11/LKB1 deficiency is proven to promote PD‐1/PD‐L1 inhibitor resistance. It also showed that genetic changes of STK11/LKB1 are the most common genetic factor of PD‐1 inhibitor resistance in KRAS‐mutant LUAD. Koyama et al.^23^ also reported that STK11/LKB1 deficiency leads to suppressed TME T‐cell activity in the lung tumor by promoting neutrophil recruitment and proinflammatory cytokine production. In sum, STK11 gene deficiency is a crucial player in TME regulation. We previously found that STK11 mutations accompanied lower macrophage M1/M2 infiltration abundance compared with STK11 non‐mutations. Therefore, this study was carried out to form a more comprehensive understanding of how STK11 mutations impact macrophage differentiation in LUAD.

Experimental results showed that STK11 mutations could promote the proliferative activity of LUAD cells and inhibit the differentiation of M1 macrophages, apoptosis, and the AMPK signaling pathway. As for the mechanism, we found that by affecting CD1E expression, STK11 mutations regulated macrophage differentiation in LUAD and hence promoted the progression of LUAD. Taken together, these findings could broaden immunotherapy strategies for patients with LUAD by shedding new insights into TAM‐targeted immunotherapy strategy.

## 2 MATERIALS AND METHODS

### 2.1 Cell culture and transfection

The human monocytic cell line THP‐1 (TIB‐202), STK11‐mutant LUAD cell lines A549 (CRM‐CCL‐185), H460 (HTB‐177), and LUAD cell lines Calu6 (HTB‐56) with nonmutant STK11 were purchased from the American Type Culture Collection. THP‐1 cells were cultured in Roswell Park Memorial Institute (RPMI) 1640 medium containing 10% fetal bovine serum (FBS), 1% streptomycin and penicillin, and 0.05 nM β‐mercaptoethanol. THP‐1 cells were induced to differentiate into macrophages using 50 ng/mL phorbol‐12‐myristate 13‐acetate (PMA). After 48 h of incubation with PMA, THP‐1 cells were collected for subsequent experiments.^24^ To investigate the effect of STK11 mutation on macrophage polarization, with STK11 siRNA (si‐STK11) and its negative control (si‐NC) obtained from RiboBio, the STK11 mutant Calu6 cell model was constructed and named Calu6STK11^KD^, and cells transfected with negative control as the control group were named Calu6STK11^WT^. With STK11 overexpression plasmid (oe‐STK11) and negative control plasmid (oe‐NC) obtained from BrainVTA, the A549 cell model transfected with oe‐STK11 with a normal STK11 expression was named as A549STK11^RES^. Cells transfected with the negative control oe‐NC were used as controls and named A549STK11^MUT25^. CD1E overexpression plasmid (oe‐CD1E) as well as negative control plasmid (oe‐NC) were obtained and transfected into Calu6STK11^KD^ and Calu6STK11^WT^ cells. After 48 h of culture, oe‐CD1E‐Calu6STK11^KD^, oe‐NC‐Calu6STK11^KD^ cells, oe‐CD1E‐Calu6STK11^WT^, and oe‐NC‐Calu6STK11^WT^ cells were collected for following experiments. In this study, cells were transfected using Lipofectamine 2000™ kit (Invitrogen) and cultured in a 5% CO_2_ incubator at 37°C.

### 2.2 Coculture of LUAD cells and macrophages

Calu6STK11^KD^ versus Calu6STK11^WT^ cells were diluted to 1.5 × 10^5^ cells/mL in RPMI‐1640 medium containing 10% FBS. Subsequently, THP‐1 cells were induced to differentiate into macrophages using PMA, and activated macrophages were cocultured with Calu6STK11^KD^, Calu6STK11^WT^ cells, oe‐CD1E‐Calu6STK11^KD^ and oe‐NC‐Calu6STK11^KD^ cells, and oe‐CD1E‐Calu6STK11^WT^ and oe‐NC‐Calu6STK11^WT^ cells at 37°C for 4 h. Culture supernatants were then collected for determination of relevant parameters.

### 2.3 Assessment of cell activity

Cell viability of LUAD cells was analyzed using a CCK‐8 kit (Med Chem Express). After seeding cancer cells at a density of 5 × 10^3^ cells per well in 96‐well plates, 10 μL CCK8 reagent was added to each well after 1, 2, 3, and 4 days of culture and the incubation continued for 2 h. The optical density of each well was detected at 450 nm using a microplate reader.

### 2.4 Quantitative reverse transcription polymerase chain reaction.(qRT‐PCR) assay

After extracting total RNA from LUAD cells using Trizol (Invitrogen) reagent, it was reverse‐transcribed into cDNA with a PrimeScript RT reagent kit (TakaRa). Subsequently, qRT‐PCR was performed on an ABI 7300 instrument (Applied Biosystem) using iQ SYBR Green Supermix kit (BIO‐RAD). Relative expression of the STK11 gene was normalized to β‐actin. Primer sequences used are listed in Table 1.

**Table 1:** Primer sequences for qRT‐PCR.

Gene	Sequence (5′ → 3′)	
STK11	Forward primer	ACGGTGCCCGGACAGG
	Reverse primer	CTGTGCCGTTCATACACACG
CD1E	Forward primer	GCTCCCCAGGCTCTACAATC
	Reverse primer	TCCAGGGTTGCTCGGAGATA
β‐actin	Forward primer	ATGACTTAGTTGCGTTACACCCT
	Reverse primer	TGCTCGCTCCAACCGACTG

### 2.5 Western blot

Total proteins were isolated from cells using RIPA lysis buffer. After loading 25 μg cellular protein samples on SDS‐PAGE gels, proteins of the same weight from each group were separated and transferred to PVDF membranes. Subsequently, membranes were blocked with 5% skimmed milk for 1 h at room temperature and later incubated with primary antibodies overnight at 4°C. The main antibodies include rabbit anti‐human CD1E antibody (1: 1000, ab187157, Abcam), rabbit anti‐human STK11 antibody (1: 1000, ab138386, Abcam), rabbit anti‐human Bax antibody (1: 1000, ab32503, Abcam), rabbit anti‐human Bak antibody (1: 10000, ab32371, Abcam), rabbit anti‐human Bcl‐2 antibody (1: 1000, ab32124, Abcam), rabbit anti‐human AMPK antibody (1: 1000, ab207442, Abcam), rabbit anti‐human p‐AMPK antibody (1: 1000, ab133448, Abcam), and rabbit anti‐human β‐actin antibody (ab115777, Abcam). Following incubation with primary antibodies, they were incubated with secondary antibody IgG (1: 2000, ab97051, Abcam) for 1 h at room temperature. Protein bands were detected with enhanced chemiluminescence reagents (Merck Millipore). β‐actin was used as an internal reference.

### 2.6 Flow cytometry analysis of macrophage phenotypes

Macrophage surface markers were analyzed by flow cytometry.^25^ In brief, coculture supernatants were collected and washed with 1% FBS in phosphate buffer saline (PBS). The cocultured cells were prepared as single‐cell suspensions and adjusted to a cell concentration of 1 × 10^6^ cells/100 μL PBS. Cells were stained with relevant antibodies: anti‐CD68‐FITC and anti‐CD86‐APC (Abcam).^26^ All cells were incubated with antibodies for 20 min at 4°C in the dark and washed twice in PBS. After analyzing cells by flow cytometry, the results were analyzed using FlowJo software.

### 2.7 Mouse model construction

Treated Calu6 cells (1 × 10^7^ cells) were suspended in 200 μL PBS and injected subcutaneously into both flanks of 4−6‐week‐old BALB/c mice (five mice per group). The size and volume of tumors were observed every 5 days. After 30 days, mice were killed to measure the maximum and minimum length (L) and weight (W) of tumors. Tumor volume was calculated as ½ LW2. The animal experiments were approved by Zhejiang Yingyang Pharmaceutical Research and Development Center Experimental Animal Ethics Committee.

### 2.8 Immunohistochemistry

Paraffin‐embedded tumor tissues were sliced and deparaffinized for gradient dehydration. Subsequently, placed in a staining jar containing EDTA, the slides underwent antigen retrieval performed by microwave heating. After dropping endogenous peroxidase blockers on the slides after cooling down, the slides were left at room temperature for 10 min. They were first incubated with primary antibodies, namely STK11 (1: 100, ab138386, Abcam), CD1E (1: 50, ab187157, Abcam), and Ki67 (1: 2000, ab15580, Abcam) for 1 h at 37°C and then for another 30 min with secondary antibodies at room temperature. 3,3′Diaminobenzidine (DAB) staining was performed using Rabbit Specific horseradish peroxidase/DAB (avidin‐biotin‐pcroxidase complex method) and Detection IHC Kit (ab64261, Abcam) according to the manual. After being counterstained with hematoxylin, the slides were mounted, followed by observing and photographing under the microscope.

### 2.9 Immunofluorescence

Paraffin‐embedded tumor tissue sections were first stained with anti‐CD68 (1: 50, ab283654, Abcam) and CD86 (1: 100, ab239075, Abcam) antibodies, and then Alexa Fluor® 488‐conjugated antibody goat anti‐rabbit IgG (1: 200, ab150077, Abcam). DAPI (Invitrogen) was added to counterstain the nuclei. After observation, pictures were taken under a fluorescence microscope and analyzed using ImagePro software.^24^

### 2.10 Statistical analysis

GraphPad Prism8.0 was used to analyze the results. Differences between two groups were analyzed using two‐sample t‐tests and between more than two groups using one‐way analysis of variance. Differences were considered statistically significant if *p* < .05.

## 3 RESULTS

### 3.1 Effect of mutant STK11 on LUAD progression

**Figure 1:**
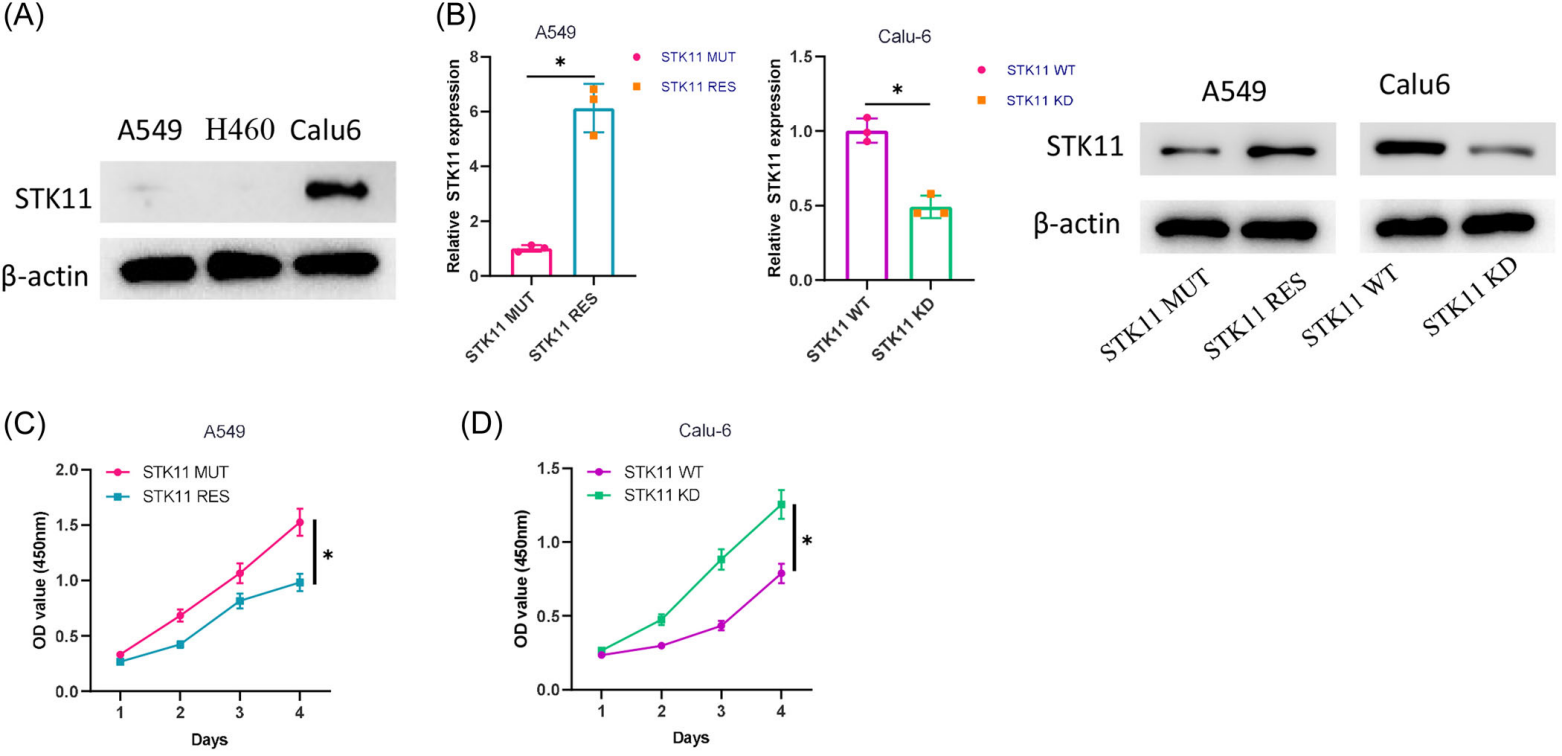
STK11 mutation induces malignant progression of LUAD. (A) STK11 expression in LUAD cells; (B) STK11 expression levels in LUAD cells after transfection were detected by qRT‐PCR and western blot; (C, D) Effect of STK11 mutation on the viability of LUAD cells after transfection; *p < .05. LUAD, Lung adenocarcinoma; qRT‐PCR, quantitative reverse transcription polymerase chain reaction; STK11, serine/threonine protein kinase 11.

STK11‐mutant NSCLC patients are confirmed with a deficiency of STK11 expression.^27^ Therefore, STK11 expression in STK11‐mutant LUAD cells (A549, H460) and LUAD cells without STK11 mutations (Calu6) was assessed by western blot. Our results that STK11 expression was significantly lower in STK11‐mutant LUAD than that in Calu6 cells provided evidence in support of the previous observation (Figure 1A). Subsequently, how mutant‐STK11 impacts the malignant behavior of LUAD cells was assessed by a cell model in vitro. A549 cells with normal STK11 expression (A549STK11^RES^), A549 cells with STK11 mutation (A549STK11^MUT^), Calu6 cells with STK11 mutation (Calu‐6STK11^KD^), and Calu6 cells with normal STK11 expression (Calu‐6STK11^WT^) were all constructed. STK11 expression was assessed across different cell models by qRT‐PCR and western blot. According to the results, STK11 expression was significantly higher in A549 cells with normal STK11 expression than in STK11‐mutated A549 cells; STK11 expression was significantly lower in STK11‐mutant Calu6 cells than in Calu6 cells with normal STK11 expression (Figure 1B). Subsequently, according to CCK‐8 results, lower cell viability was observed in A549 cells with normal STK11 expression, yet STK11‐mutant Calu6 cells are found with significantly higher cell activity compared with Calu6 cells with normal STK11 expression (Figure 1C,D). STK11 is a key kinase in the AMPK signaling pathway.^28^ Then, we detected the protein expression levels of AMPK/p‐AMPK in cells after transfection with STK11 knockdown, and the results showed that the protein expression level of AMPK showed no significant change after STK11 knockdown, but the protein expression of p‐AMPK was decreased significantly (Supporting Information: Figure 1A). Based on the above observation, STK11 was supposed to induce the malignant progression of LUAD.

### 3.2 Effect of STK11 mutation on macrophage differentiation in LUAD

**Figure 2:**
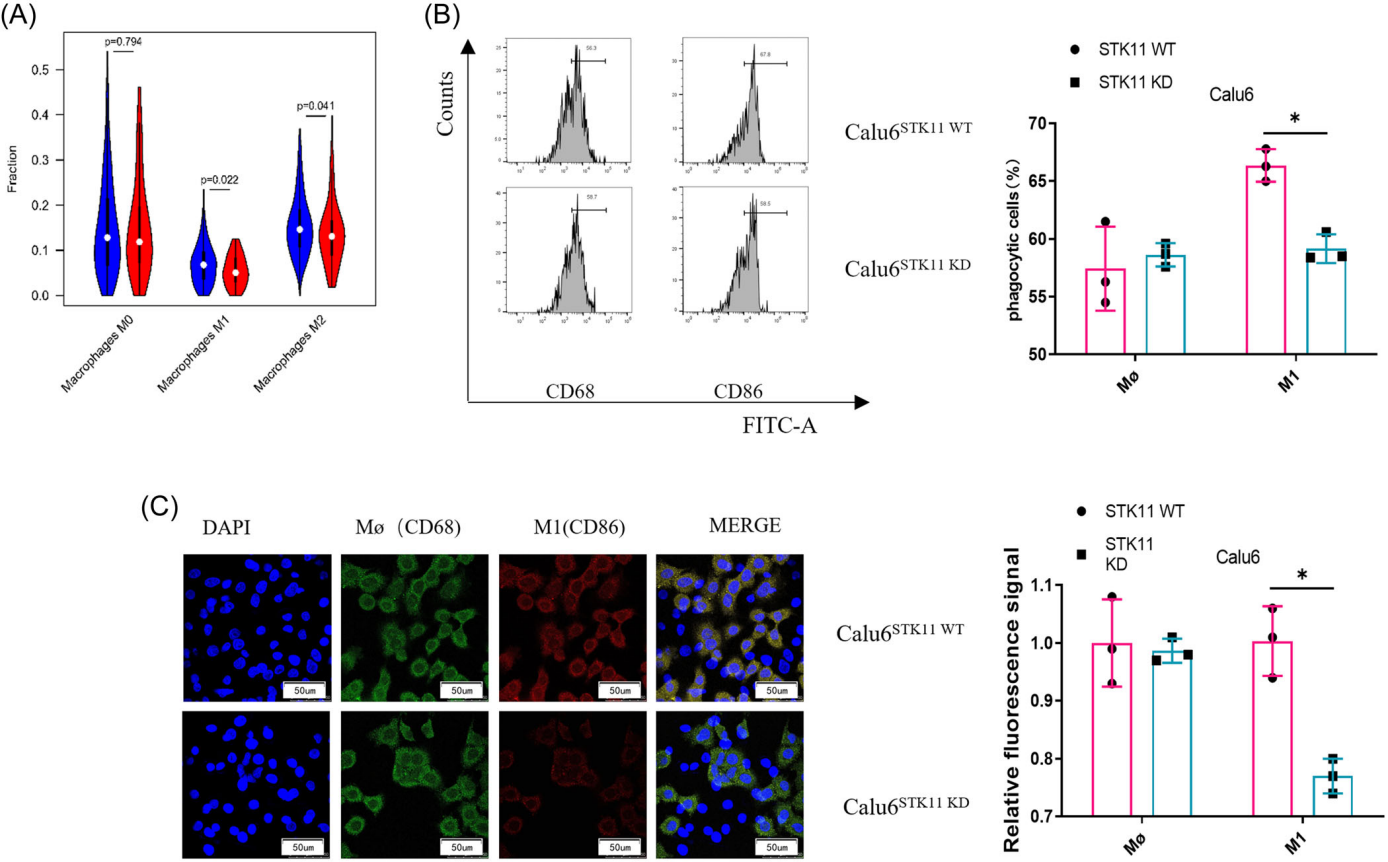
Effect of STK11 mutation on macrophage differentiation in LUAD. (A) Bioinformatics analysis of macrophage infiltration in LUAD tumor tissues with normal STK11 and deficient STK11 expression; blue indicates LUAD tissue with normal STK11 expression, red indicates LUAD tissue with STK11 mutation; (B) Effect of STK11 mutation on macrophage differentiation after transfection were detected by flow cytometry; (C) Expression of M0 and M1 macrophage marker proteins in LUAD cells after transfection was detected by immunofluorescence; *p < .05. LUAD, Lung adenocarcinoma; qRT‐PCR, quantitative reverse transcription polymerase chain reaction; STK11, serine/threonine protein kinase 11.

Previous research suggested that STK11 mutation affects the level of immune cell infiltration in the TME.^29^ To investigate the regulatory role of STK11 in the TME of LUAD, immune cell infiltration of LUAD tumor tissue samples with STK11 mutation and normal STK11 expression was analyzed by bioinformatics. The results indicated that M1 and M2 macrophages were significantly decreased in STK11 mutation samples (Figure 2A). The observation was validated by another in vitro cell model.

STK11 mutant (Calu6STK11^KD^) and wild‐type (Calu6STK11^WT^) LUAD cell lines were cocultured with PMA‐activated THP‐1 cells. Macrophage differentiation was detected using flow cytometry. The results found more M1 macrophages in the coculture system of Calu6 with normal STK11 expression and THP‐1; however, fewer M1 macrophages were found in the coculture system of STK11‐mutant Calu6 and THP‐1 (Figure 2B). Subsequently, M0 and M1 macrophage markers were further detected in the two coculture systems by immunofluorescence. A lower CD86 expression was found in the coculture system of STK11‐mutant Calu6 cells and THP‐1 (Figure 2C). All these results suggested that the STK11 mutation curbed the differentiation of immature macrophages into the M1 type in LUAD.

### 3.3 STK11 mutation impacts CD1E expression to regulate the differentiation of macrophages in LUAD

**Figure 3:**
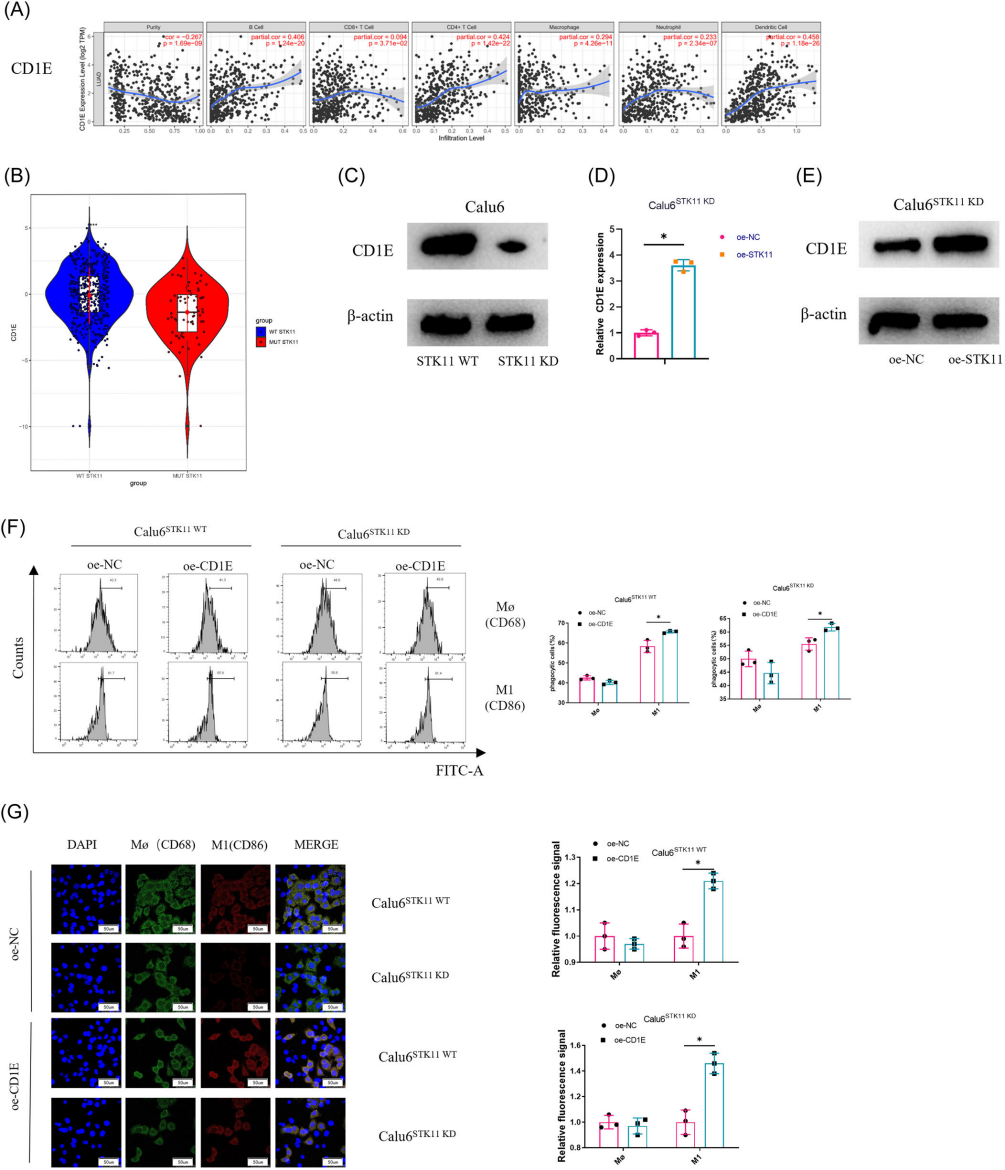
STK11 mutation affects CD1E expression to regulate macrophage differentiation in LUAD. (A) Bioinformatics analysis of the correlation between the signature gene (CD1E) expression and immune infiltration in STK11‐mutant LUAD patients; (B) Bioinformatics analysis of CD1E expression in STK11‐mutant and STK11‐nonmutant LUAD tumor tissues; (C) CD1E expression in STK11‐mutant and STK11‐nonmutant LUAD tumor cells; (D, E) CD1E expression in STK11‐mutant and STK11‐nonmutant LUAD tumor cells after overexpression of STK11; (F) Effect of CD1E overexpression on macrophage differentiation in LUAD after transfection was detected by flow cytometry; (G) M0 and M1 macrophage marker proteins expression in LUAD cells after transfection was detected by immunofluorescence; *p < .05. LUAD, Lung adenocarcinoma; qRT‐PCR, quantitative reverse transcription polymerase chain reaction; STK11, serine/threonine protein kinase 11.

To unveil the molecular mechanism involved in the regulation of macrophage differentiation in STK11‐mutant LUAD, the association between immune cell infiltration and the signature gene expression was analyzed by bioinformatics. The results found CD1E expression in a strong positive correlation with B‐cell, CD4^+^ T‐cell, macrophage, and dendritic cell infiltration (Figure 3A). In addition, CD1E was notably downregulated in LUAD patients with STK11 mutation (Figure 3B). Subsequently, in vitro experiment was conducted for validation. According to the western blot results, CD1E protein expression was significantly downregulated in STK11‐mutated LUAD cells compared with its LUAD peer without STK11 mutations (Figure 3C). The effect of STK11 overexpression on CD1E expression was later investigated. According to the qRT‐PCR and western blot results, overexpressed STK11 could increase CD1E mRNA and protein expression in STK11‐mutated LUAD cells, compared with the control group (Figure 3D,E). Subsequently, the effect of CD1E overexpression on macrophage differentiation in STK11‐mutant LUAD was investigated by coculturing PMA‐activated THP‐1 cells with STK11‐mutant/wild‐type LUAD cells with overexpressed CD1E respectively. Macrophage differentiation was detected by flow cytometry, whose results showed increased M1 macrophages in the coculture system of oe‐CD1E‐Calu6STK11‐KD and THP‐1 compared with the control group. Besides, as for the STK11 nonmutant coculture system, overexpression of CD1E significantly boosted the quantity of M1 macrophages (Figure 3F). Immunofluorescence results showed that overexpressed CD1E facilitated CD86 expression in the STK11‐nonmutant coculture system, while significantly increasing the expression of CD86 in the STK11‐mutant coculture system (Figure 3G). Taken together, the results indicated that STK11 mutation would lead to downregulation of CD1E, which in turn inhibited macrophage differentiation to the M1 type in LUAD.

### 3.4 Effect of STK11 mutation on LUAD tumor growth validated in vivo

**Figure 4:**
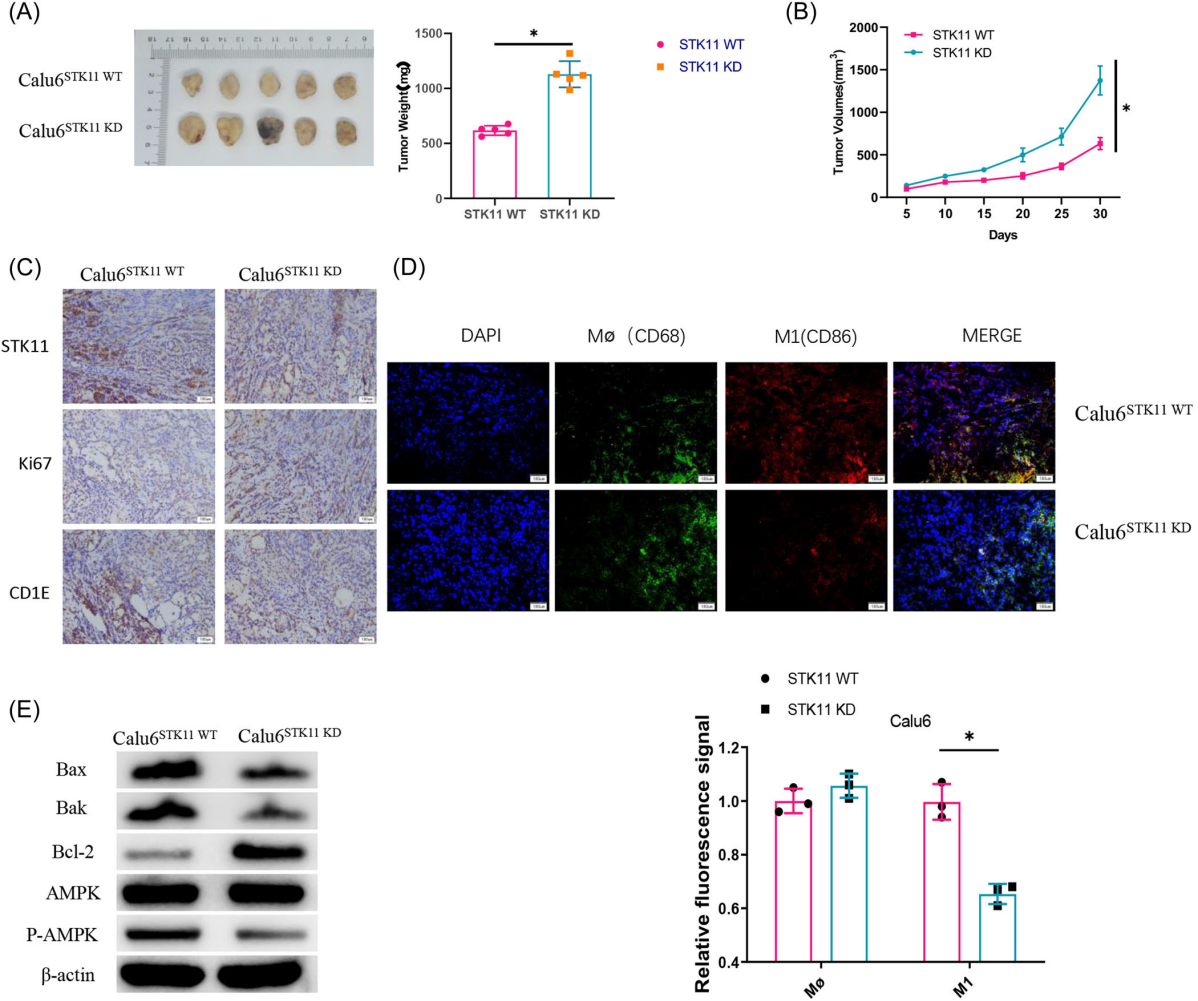
Effect of STK11 mutation on LUAD tumor growth validated in vivo. (A) Tumor morphology and weight of mice; (B) Tumor volume of mice; (C) Expression of STK11, Ki67, and CD1E in mouse tumor tissues after transfection; (D) Immunofluorescence staining of CD86 and CD68 in mouse tumor tissues after transfection; (E) Expression levels of Apoptosis markers and AMPK signaling pathway marker proteins were detected by western blot after transfection treatment; *p < .05. LUAD, Lung adenocarcinoma; qRT‐PCR, quantitative reverse transcription polymerase chain reaction; STK11, serine/threonine protein kinase 11.

To confirm the results of in vitro cell experiments, mouse LUAD models stably transfected with Calu6STK11^WT^ and Calu6STK11^KD^ cells were first constructed. As we kept records of tumor size, volume, and weight, it was discovered that both tumor volume and weight were significantly increased in mice injected with Calu6STK11KD cells compared with their control counterparts (Figure 4A,B). Subsequently, immunohistochemistry was used to detect the expression of STK11, Ki67, and CD1E in tumor tissues. The results found significant downregulation of STK11 and CD1E, and significant upregulation of Ki67 in mice injected with Calu6STK11^KD^ cells (Figure 4C). After that, immunofluorescence was used to detect macrophage infiltration in mouse tumor tissues. It was discovered that CD86 was significantly fewer in mouse tumor tissues injected with Calu6STK11^KD^ cells compared with the counterpart (Figure 4D). Then, western blot was used to test the protein expression of apoptosis and AMPK/p‐AMPK. The results showed that after knocking down STK11, the protein expression levels of Bax and Bak were significantly reduced, while the protein expression of Bcl‐2 was significantly increased. At the same time, the protein expression of p‐AMPK was significantly reduced after knocking down STK11, but there was no significant change in the protein expression of AMPK. The observation suggested that STK11 mutation reduced cellular infiltration in M1 macrophages, apoptosis, and the AMPK signaling pathway that promoted tumor growth. In short, STK11 mutation promoted tumor growth in mice.

## 4 DISCUSSION

STK11 (LKB1), a tumor suppressor gene originally identified in Peutz‐Jeghers syndrome was found to be involved in the cell cycle and TP53‐induced apoptosis. Apart from that, STK11 is associated with the Wnt signaling and TGF‐β signaling pathways, through which, STK11 participates in cell proliferation, cell polarity regulation, and lipid and glucose metabolism regulation.^30^, ^31^, ^32^, ^33^ As one of the most frequently inactivated tumor suppressors in NSCLC,^23^ how mutated STK11 is involved in tumor progression has been covered in many studies. According to previous studies, decreased and inactivated STK11 expression is associated with epithelial‐mesenchymal transition (EMT). For instance, STK11 inactivation triggers EMT in lung cancer cells by inducing ZEB1 expression.^34^ Decreased STK11 expression is associated with EMT in gastric cancer and hence poor prognosis.^35^ This study confirmed that mutated STK11 could promote the proliferation of LUAD cells. In addition, STK11 mutation is associated with tumor therapy resistance. For example, LKB1 deficiency promotes resistance to radiotherapy in NSCLC patients. The process depends on the KEAP1/NRF2 pathway for redox homeostasis. Suppression of the KEAP1/NRF2 pathway via KEAP1 expression, or pharmacologic blockade of glutaminase (GLS) 1 sensitized LKB1‐deficient tumors to radiotherapy.^36^ The study conducted by Pore et al.^37^ found a link between STK11 mutations and resistance to the anti‐PD‐L1 antibody durvalumab (alone/in combination with the anti‐CTLA4 antibody tremelimumab), which may check the response to checkpoint inhibitors by inhibition of bone marrow cell biology and can reverse this resistance by knocking down STAT3. LKB1 knockdown induces AMPK inactivation and attenuation of antigen presentation, thus promoting the immune escape of lung cancer cells.^38^ Consistent with the above trend, we found that STK11 knockdown inhibited p‐AMPK protein expression, and the same trend was also observed in the mouse model. Therefore, STK11 mutation is supposed to be the crucial driver of cancer progression and resistance to tumor therapy. This study attempted to shed new insights into the role mutated STK11 played in LUAD progression.

Recent studies confirmed that oncogene mutations alter immune infiltration of immune cells in the TME, which in turn impacts tumor progression.^39^ More importantly, STK11 mutations are involved in immune cell regulation in the TME. As Koyama et al.^23^ investigated the consequence of STK11/LKB1 deficiency on the TIME in a mouse model of KRAS‐driven NSCLC, and they confirmed that STK11/LKB1 deficiency promotes neutrophil recruitment and proinflammatory cytokine production to suppress T‐cell activity in the lung TME. In this study, a link between STK11 mutations and decreased M1 macrophages was found in LUAD tumor tissues. Previously, M1 macrophages were found to exert an inhibitory role on tumor growth. Similarly, higher LUAD cell viability and increased tumor size were also found in STK11‐mutant cells and mouse models. Besides, by analyzing samples from LUAD patients with STK11 mutations, this study suggested that STK11 may affect LUAD progression by affecting the differentiation and infiltration of M1 macrophages in tumor tissue. This finding was consistent with less infiltration of immune cells found in STK11‐deficient LUAD tumors, including dendritic cells, neutrophils macrophages, CD4^+^ T cells, B cells, and CD8^+^ T cells.^29^ Low expression of CD1E was also found in STK11 mutant LUAD patients, suggesting that STK11 mutation may affect the expression of CD1E to inhibit the immune infiltration of M1 macrophages in LUAD. In summary, understanding how STK11 mutation influences tumor immunity is crucial to the development of effective individualized treatments for LUAD.

Our study validated that mutations of tumor‐inhibiting genes could also impact the TIME of lung cancer. This study demonstrated that STK11 losses suppressed immune infiltration of M1 macrophages in LUAD, which in turn promoted the malignant progression of LUAD. Our results also suggested that, by downregulating CD1E, STK11 deficiency may inhibit the differentiation of M1 macrophages in LUAD and then promote tumor growth. Instead, an enhanced CD1E expression would result in increased M1 macrophages. These findings not only added evidence to the potential of CD1E as a therapeutic target for STK11‐deficient lung cancer but also to a novel immunotherapeutic strategy for LUAD patients that targets immune cells in the TIME. Unfortunately, this study still has some limitations. Clinical research about the regulatory network of STK11 mutation in the progression of LUAD will be completed in the future. The effect of STK11 mutation on the response to immunotherapy in LUAD will also be further explored. In conclusion, our study provided new ideas for the development of individualized immunotherapy based on improving macrophage polarization in STK11‐mutated patients.

## AUTHOR CONTRIBUTION

**Qingfeng Zhang**: Conceptualization (lead); writing—original draft (lead); **Juan Feng**: Formal analysis (lead); collection and assembly of data (equal). **Kui Liu**: Software (lead); Writing—review and editing (lead). **Xiaoyan Yang**: Methodology (lead); collection and assembly of data (equal). **Yun Huang**: Conceptualization (supporting); writing—original draft (supporting). **Bo Tang**: Writing—original draft (supporting); collection and assembly of data (supporting).

## CONFLICT OF INTEREST STATEMENT

The authors declare no conflict of interest.

## ETHICS STATEMENT

The study was approved by Zhejiang Yingyang Pharmaceutical Research and Development Center Experimental Animal Ethics Committee. The methods were carried out in accordance with the approved guidelines.

## Supporting information


**Supplementary Figure 1 Expression of AMPK signaling pathway markers**. (A) The expression of AMPK signaling pathway markers after transfection was detected by western blot.

## Data Availability

The data in this study are available from the corresponding author on reasonable request.
