# Supplementary figures and images for: STK11 mutation impacts CD1E expression to regulate the differentiation of macrophages in lung adenocarcinoma
Source: Immun Inflamm Dis. 2023 Jul 27;11(7):e958. doi: 10.1002/iid3.958 (PMC10373563; doi:10.1002/iid3.958)

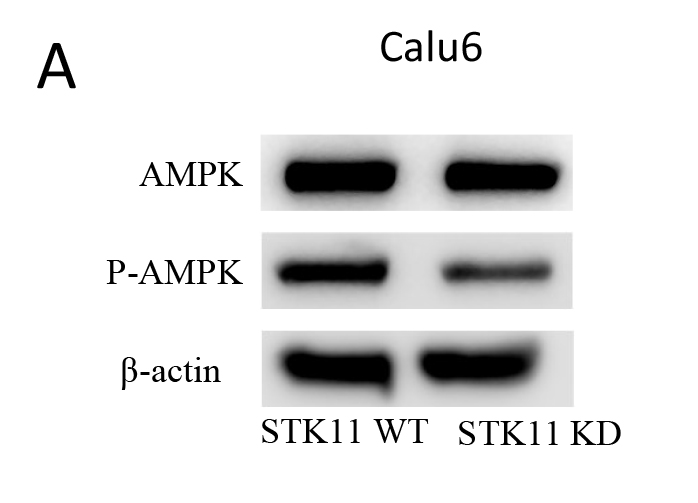

Supplement: Supplementary file 1 — Supplementary Figure 1 Expression of AMPK signaling pathway markers. (A) The expression of AMPK signaling pathway markers after transfection was detected by western blot. [file IID3-11-e958-s001.jpg]
